# Supplementary material for: Ordered creation and motion of skyrmions with surface acoustic wave
Source: Nat Commun. 2023 Jul 22;14:4427. doi: 10.1038/s41467-023-40131-1 (PMC10363109; doi:10.1038/s41467-023-40131-1)
Supplement: Supplementary file 1 — Supplementary Information [file 41467_2023_40131_MOESM1_ESM.pdf]

Supplementary Information

Ordered creation and motion of skyrmions with surface acoustic wave

Ruyi Chen, Chong Chen, Lei Han, Peisen Liu, Rongxuan Su, Wenxuan Zhu, Yongjian Zhou, Feng Pan, Cheng Song

Key Laboratory of Advanced Materials (MOE), School of Materials Science and Engineering, Beijing Innovation Center for Future Chip, Tsinghua University, Beijing 100084, China

Supplementary Note 1: Micromagnetic simulations of SAW induced ordered alignment of skyrmions

Micromagnetic simulations were performed on the Object Oriented MicroMagnetic Framework (OOMMF) with SAW and temperature modules included[1]. The magnetic dynamics are described by the LLG equation:

$$\frac{d\boldsymbol{m}}{dt} = -|\gamma|\boldsymbol{m} \times \boldsymbol{H}_{eff} + \alpha(\boldsymbol{m} \times \frac{d\boldsymbol{m}}{dt})$$

Here, γ is the gyromagnetic ratio, α is the Gilbert damping coefficient, and $\boldsymbol{H}_{eff} = -\partial H_{total}/\partial \boldsymbol{m}$ illustrate the effective magnetic field of the total Hamiltonian H_{total}, which can be expressed as

$$H_{total} = -A_{intra}\sum_{(i,j)}\boldsymbol{m}_i \cdot \boldsymbol{m}_j + D\sum_{(i,j)}\left(\boldsymbol{v_{ij}} \times \hat{\boldsymbol{z}}\right) \cdot \boldsymbol{m}_i \times \boldsymbol{m}_j + K\sum_i(1 - m_{i,z}^2) + H_{\text{DDI}} + H_{\text{SAW}} + H_{zeeman} + H_{\text{T}}$$

Where $\boldsymbol{m}_{\mathbf{i}}$ ($\boldsymbol{m}_{\text{j}}$) is the local magnetic moment for the site *i*. $\boldsymbol{v}_{\mathbf{ij}}$ and $\hat{\boldsymbol{z}}$ denote the direction from site *i* to site *j* and the film normal direction. A_{intra}, *D*, and *K* are the intralayer ferromagnetic (FM) exchange constant, the DMI constant, and the PMA constant, respectively. H_{DDI} gives the dipole-dipole interaction. H_{SAW} represents energy carried by SAW, which is written by $E = b_1\varepsilon_{xx}m_x^2 + b_1\varepsilon_{zz}m_z^2 + 2b_2\varepsilon_{xz}m_xm_z$, where the strain matrix is reading as follow[2,3]:

$$\begin{pmatrix} \varepsilon_{xx} & 0 & \varepsilon_{xz} \\ 0 & 0 & 0 \\ \varepsilon_{xz} & 0 & \varepsilon_{zz} \end{pmatrix} = \begin{pmatrix} \varepsilon_{xx}^0 coskx\ \ cos\omega t & 0 & -\varepsilon_{xz}^0 sinkx\ \ cos\omega t \\ 0 & 0 & 0 \\ -\varepsilon_{xz}^0 sinkx\ \ cos\omega t & 0 & \varepsilon_{zz}^0 coskx\ \ cos\omega t \end{pmatrix}$$

Where k and ω are the wave vector and the angular frequency of the SAW, respectively. H_{zeeman} represents the Zeeman energy under magnetic field. H_T gives the temperature-dependent Hamiltonian with corresponding thermal Gaussian stochastic magnetic field $\boldsymbol{h}_{t,i}(x,t)$ satisfying $< \boldsymbol{h}_{T,i}(\boldsymbol{x},\boldsymbol{t})\boldsymbol{h}_{T,j}(\boldsymbol{x}',\boldsymbol{t}') >= \frac{2k_B T\alpha}{\hbar}\boldsymbol{a}^2\boldsymbol{\delta}(\boldsymbol{x}-\boldsymbol{x}')\boldsymbol{\delta}_{ij}\boldsymbol{\delta}(\boldsymbol{t}-\boldsymbol{t}')$[4]. T denotes the temperature, k_B is the Boltzmann constant, a is the area of the lattice, $\hbar$ is the reduced Planck constant, and δ_{ij} is the Kronecker delta function.

The parameters are set in consistence with practical situations of our films: $T = 50 - 315$ K, $D = 4.0$ mJ m^{-2},[4–6] $K = 1.2$ MJ m^{-3}, $A_{intra} = 5$ pJ m^{-1}, $M_s = 1.2$ MA m^{-1}., magnetic field 300 kA m^{-1}.[7] As for the SAW, $\omega/2\pi = 3.5$ GHz, $b_1 = b_2 = 10$ MJ m^{-3},[8,9] $\varepsilon_{xx}^0 = 0.125$, $\varepsilon_{zz}^0 = 0.0125$, and $\varepsilon_{xz}^0 = 0.00625$. The Gilbert damping coefficient $\alpha = 0.3$ and the gyromagnetic ratio $\gamma = -2.211 \times 10^5$ m A^{-1} s^{-1}. The mesh is 420 nm × 420 nm × 1.4 nm with the cellsize of 1.4 nm × 1.4 nm × 1.4 nm. For the simulation of current-driven skyrmion motion, we add spin polarized current j of 0.75×10^{11} A m^{-2} with the polarization rate of 0.4.

Supplementary Note 2: Finite element simulations of the SAW admittance and transmission spectrum

The SAW admittance and transmission spectrums are simulated by commercial COMSOL 6.0 Multiphysics platform using piezoelectricity component based on solid mechanics and electrostatics. To reduce computational load, we build 2.5D resonator and acoustic delay line (ADL) model and set a proper thickness in plane. As for resonator simulation, the model possesses following geometry: wavelength $\lambda = 20$ μm, the lithium niobate (LN) substrate was set to be 4.5λ and 1λ perfect match layer was added to the bottom for capturing losses related to bulk wave radiation. In order to simplify the calculation time, only a period of IDT is considered, and periodic boundary condition has been set in both the x- and y-directions to simulate the infinite length. As for ADL simulations, the distance of the two IDTs is 200 μm, and each IDT has 20 pairs of single-type fingers. In each IDT, the width and gap of the fingers are both set to 5 μm, defining the fundamental wavelength of the SAW to be 20 μm. Perfectly matched layers (PML) are placed around the LN cells for the same effect as resonator. The frequency domain studies are carried out to calculate the Y- and S-parameters of resonator and ADL, respectively. The calculated S_{21} of this device showing three transmission maxima, in coincidence with those of the device used in the experiment.

For the second peak which is used in our experiments, the LLSAW is excited when an RF voltage is applied to IDT 1 (Fig. S4a). Then, LLSAW propagates along the +x-axis and coherently superimposed with the reflected wave from the IDT 2 to form a bulk longitudinal wave (leakage along the −z-axis). Meanwhile, the strain distribution on the substrate surface has the characteristics of standing waves, i.e. there are some locations

where the strain is always zero (nodes) and largest (anti-nodes), as shown in Fig. S4d. Along with these anti-nodes, the position of the skyrmion array is determined.

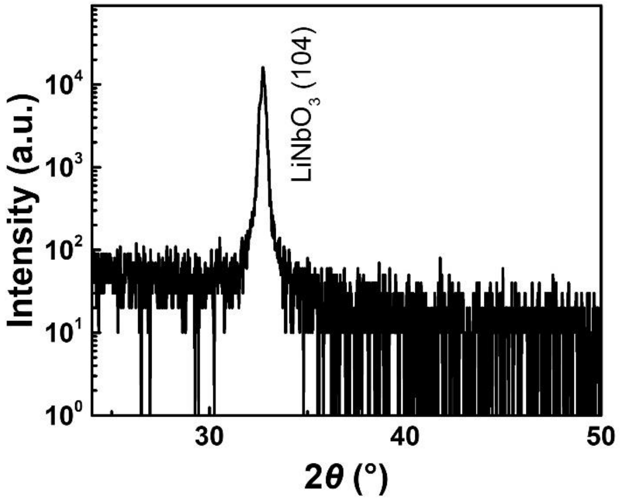

Fig. S1| XRD spectrum of the $LiNbO_3$ substrate. Only the diffraction peak of the $LiNbO_3$ (104) exist, corresponding to the 128 °, rotated, Y-cut $LiNbO_3$ substrate.

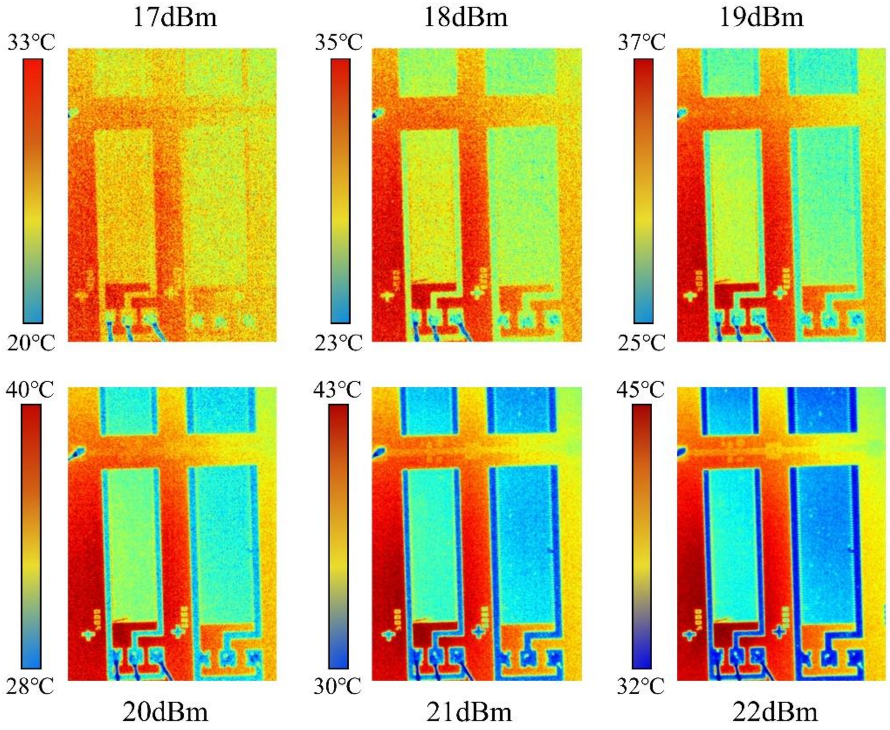

Fig. S2| Temperature distribution in SAW devices at the frequency of 365.65 MHz with different power by an infrared radiation camera. With increasing the input power from 17 dBm to 22 dBm, the thermal effect is becoming more and more obvious.

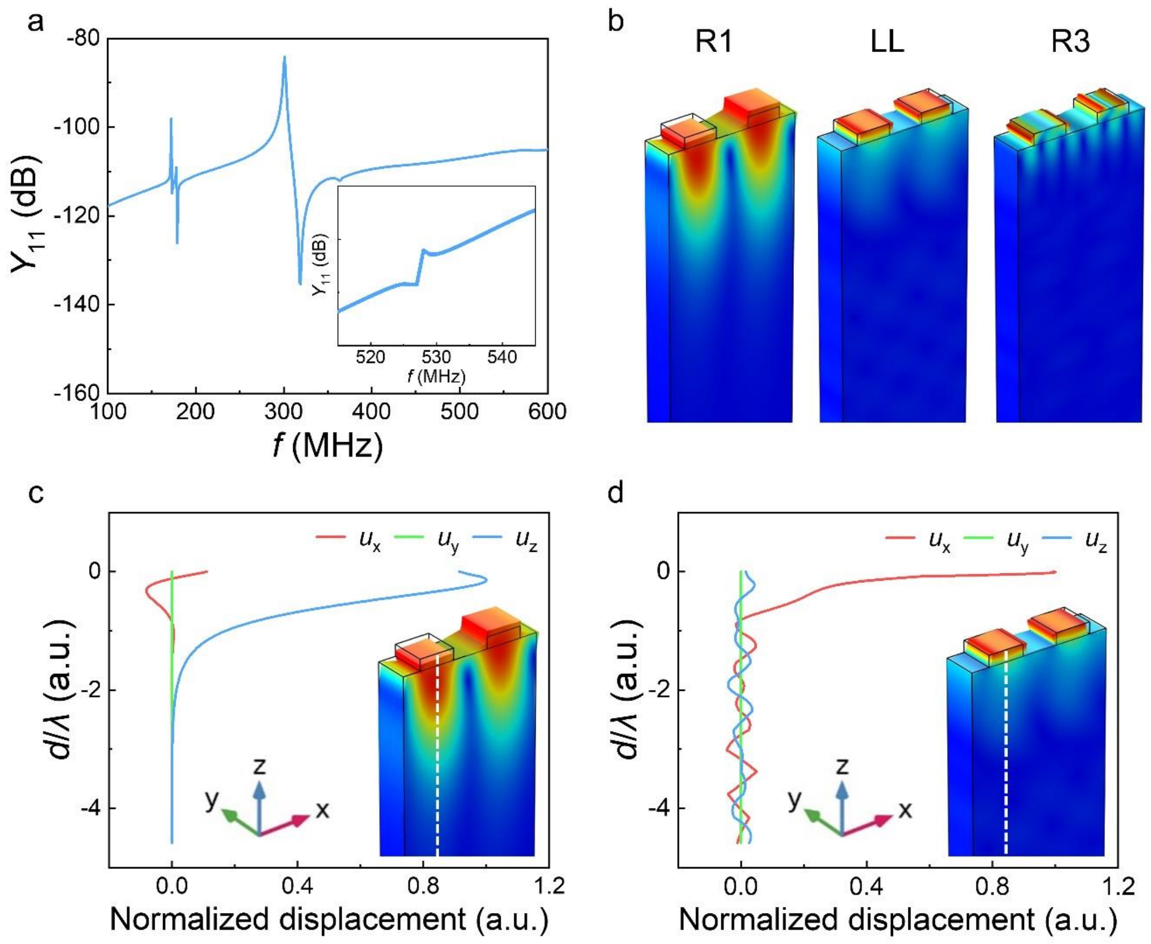

Fig. S3| Finite element simulations of the SAW modes based on 128°Y-90°X $LiNbO_3$ substrate with λ = 20 μm. **a**, The simulated device admittance curve. There are three resonance peaks with frequencies around 179, 319 and 527 MHz, respectively. The third peak is relatively weak (see the inset). **b**, The deformation shape for the three SAW modes at resonant frequency. From left to right, they are the first Rayleigh SAW (R1), the longitudinal leaky SAW (LL) and the third Rayleigh SAW (R3). **c**,**d**, The distribution of three normalized particle displacement components along the white dashed line for R1 (c) and LL (d). The insets show the local magnification of the deformation and white dotted lines extending from the electrode surface to the inside of the substrate. The

distance from the electrode surface *d* is normalized through dividing by the wavelength λ. The propagation direction of SAW is defined as the +*x*-axis. The displacements of R1 concentrate well at the surface (1 ~ 2 times of λ), while those of LL go deeply into the substrate.

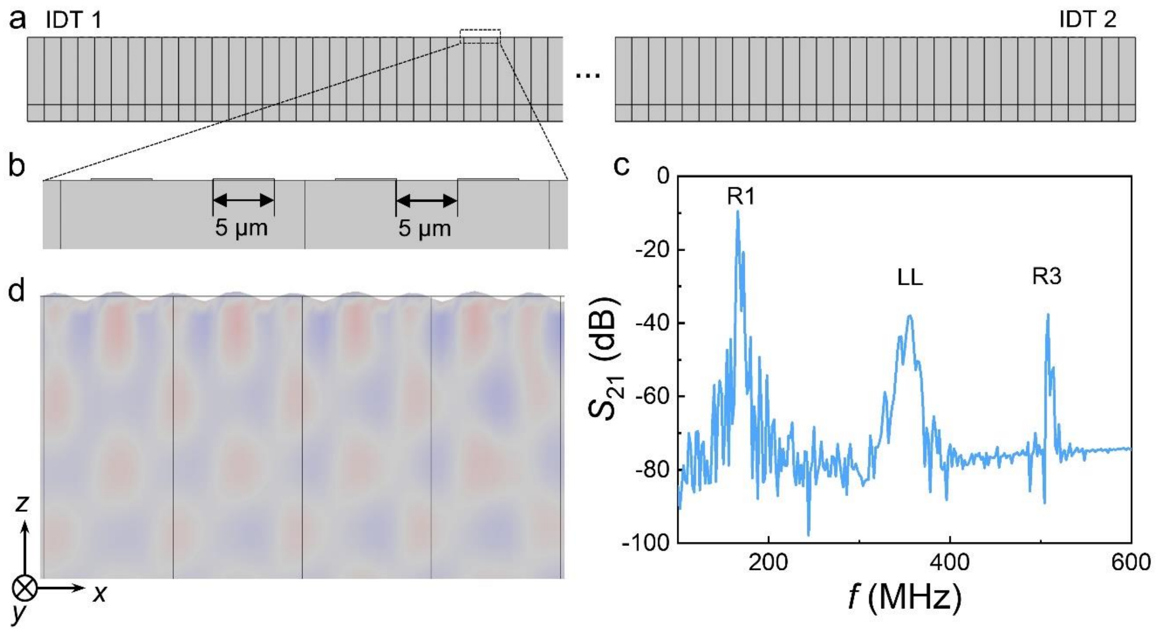

Fig. S4| Finite element simulations of the SAW transmission spectrum in a delay line based on 128° Y-90° X $LiNbO_3$ substrate. **a,b**, Device geometry and corresponding coordinate system. The distance of the two IDTs is 300 μm, and each IDT has 20 pairs of single-type fingers. In each IDT, the width and gap of the fingers are both set to 5 μm, defining the fundamental wavelength of the SAW to be 20 μm. **c**, The calculated S_{21} of this device, showing three transmission maxima, in coincidence with those of the device used in the experiment. **d,** The spatial distribution of ε_{xx} near the surface of $LiNbO_3$ substrate in the *xOz* plane, showing the characteristics of standing waves. ε_{xx} plays a leading role in the formation of ordered skyrmions.

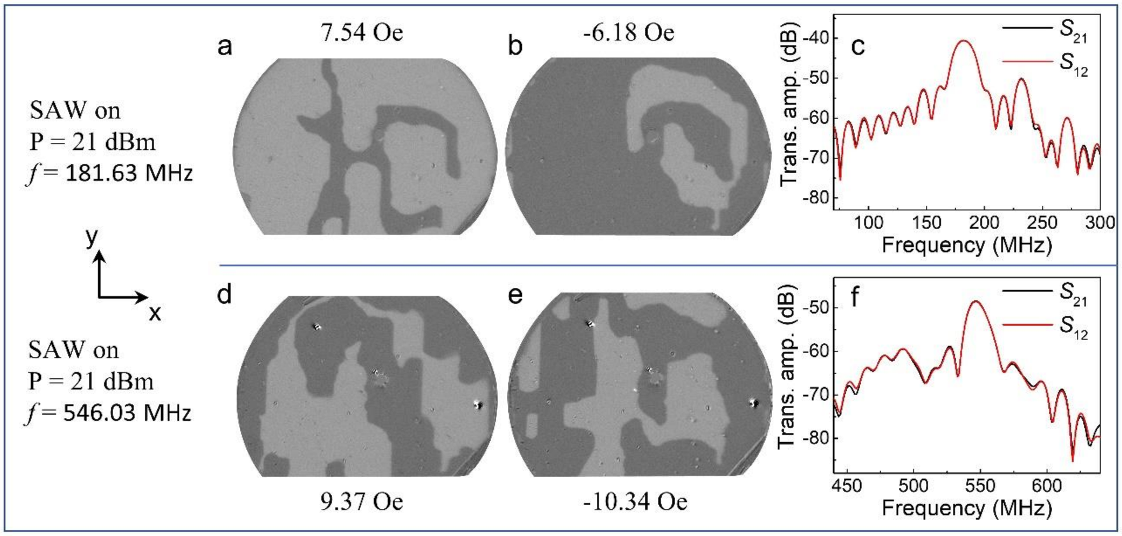

Fig. S5| Evolution process of magnetic domains with exciting surface acoustic waves at the frequency of 181.63 MHz and 546.03 MHz. **a,b**, MOKE images taken at the perpendicular magnetic field of 7.54 Oe and −6.18 Oe with applying the frequency of 181.63 MHz and power of 21 dBm. **c**, The transmission spectrum of the surface acoustic waves at the range from 70 MHz to 300 MHz. **d,e**, MOKE images taken at the perpendicular magnetic field of 9.37 Oe and −10.34 Oe with applying the frequency of 546.03 MHz and power of 21 dBm. **f**, The transmission spectrum of the surface acoustic waves at the range from 440 MHz to 640 MHz. All the spectra are obtained using radiofrequency signal of $P \sim -5$ dBm.

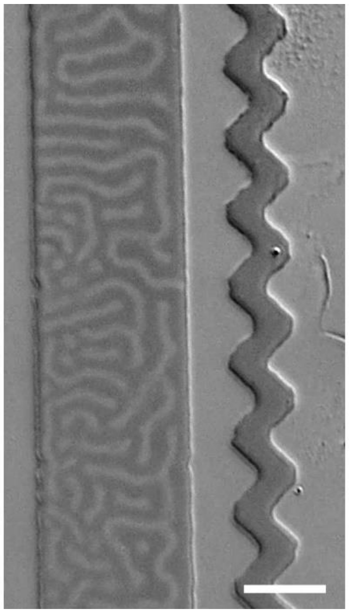

Fig. S6| MOKE image for the mixed states of skyrmions and stripe domains generated by thermal effect in the sample of Co/Pd/Co/Pd/Co/Pt. Scale bar, 10 μm.

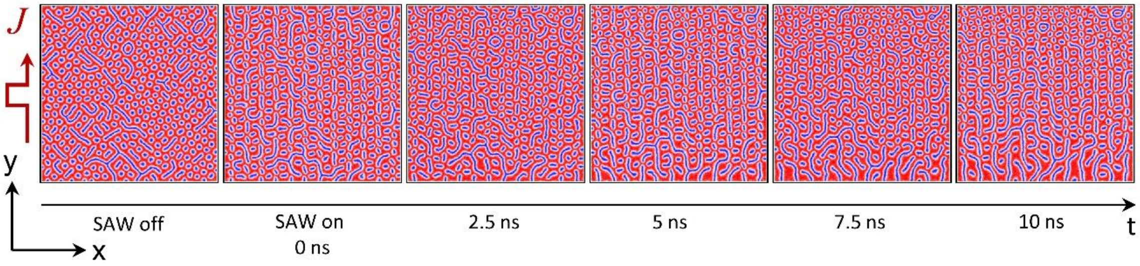

Fig. S7| Simulated current induced skyrmions motion with exciting SAWs. Similar to the discussion in the main text, skyrmions are first randomly distributed before exciting SAWs and then orderly aligned in the *y* direction once the SAW is applied. Under the current pulses, skyrmions are moving in the *y* direction nearly in a straight line with negligible skyrmion Hall effect.

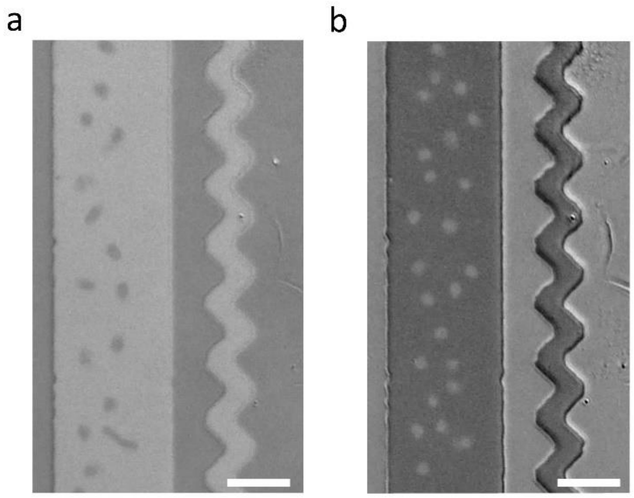

Fig. S8| MOKE images of the sample under the heating current of 2.6 mA with the perpendicular magnetic field of −1.1 Oe (**a**) and 0.9 Oe (**b**). Scale bar, 10 μm. Creation of ferromagnetic skyrmions by thermal effect by fabricating a heater on the side of the channel. The ferromagnetic sample with the structure of Co/Pd/Co/Pd/Co/Pt and a heater was fabricated to measure the skyrmion Hall effect without applying surface acoustic wave.

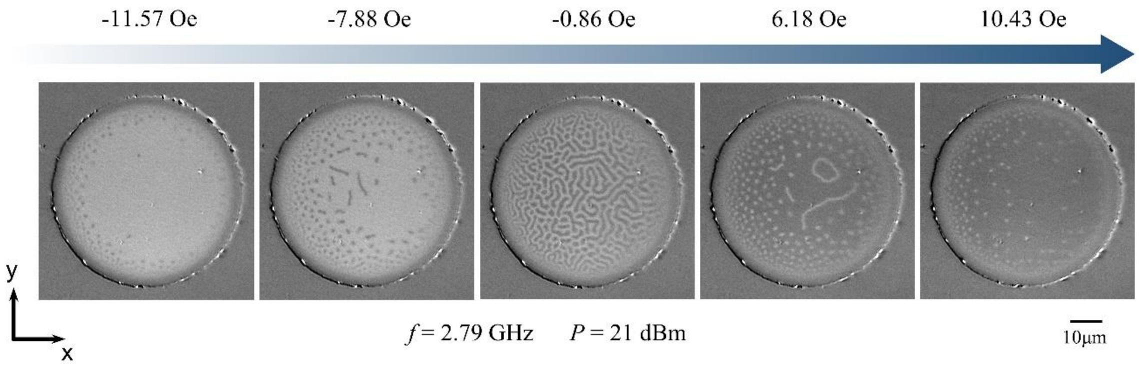

Fig. S9| Consecutive MOKE images acquired from the Co/Pd/Co/Pd/Co/Pt multilayer under different perpendicular magnetic fields with exciting SAWs of 2.79 GHz. The width and gap of the fingers are both designed as 350 nm. Images are taken at –11.57 Oe,

–7.88 Oe, –0.86 Oe, 6.18 Oe and 10.43 Oe with the radiofrequency signal of P = 21 dBm. Intertwined maze domains were observed at the magnetic field of –0.86 Oe which evolves into the mixture of stripe domains and skyrmions with the increasing of perpendicular magnetic field. Different from the organized alignment of skyrmions observed in the main text, the created skyrmions and stripe domains are randomly distributed in the device with frequency of 2.79 GHz.

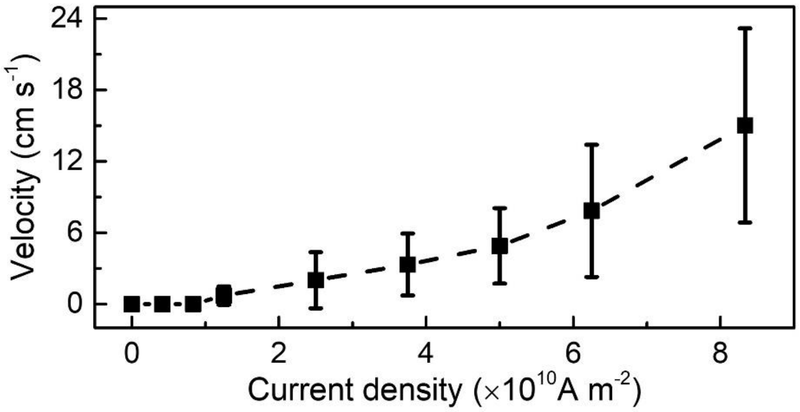

Fig. S10| Current density dependence of average velocity of skyrmion motion generated by SAWs. When the current density is small (J < 1.25 × 10^{10} A m^{-2}), skyrmions cannot be driven by the current, which is due to the fact that a certain large of current is needed to overcome the skyrmions pinning barrier. As increasing current density from 1.25 × 10^{10} A m^{-2} to 8.3 × 10^{10} A m^{-2}, skyrmions start to move and the corresponding velocity of skyrmion motion increases rapidly.

Supporting Video Captions:

Supplementary Video 1 | Simulated evolution of the magnetic domain before and after applying SAWs.

Supplementary Video 2 | Simulated current-driven motion of the skyrmions with SAWs at $j = 0.75\times10^{11}$ A m^{-2}.

Supplementary Information References:

1. Yahagi, Y., Harteneck, B., Cabrini, S. & Schmidt. H. Controlling nanomagnet magnetization dynamics via magnetoelastic coupling. *Phys. Rev. B* **90**, 140405 (2014)
2. Sasaki, R., Nii, Y., Iguchi, Y. & Y. Onose. Nonreciprocal propagation of surface acoustic wave in $Ni/LiNbO_3$. *Phys. Rev. B* **95**, 020407 (2017).
3. Shuai, J. T., Ali, M., Lopez-Diaz, L., Cunningham, J. E. & Moore, T. A. Local anisotropy control of Pt/Co/Ir thin film with perpendicular magnetic anisotropy by surface acoustic waves. *Appl. Phys. Lett.* **120**, 252402 (2022).
4. Sampaio, J., Cros, V., Rohart, S., Thiaville, A. & Fert, A. Nucleation, stability and current-induced motion of isolated magnetic skyrmions in nanostructures. *Nat. Nanotechnol.* **8**, 839−844 (2013).
5. Zhang, X., Zhou, Y. & Ezawa M. Magnetic bilayer-skyrmions without skyrmion Hall effect. *Nat. Commun.* **7**, 10293 (2016).
6. Soumyanarayanan, Anjan. et al. Tunable room-temperature magnetic skyrmions in Ir/Fe/Co/Pt multilayers. *Nat. Mater.* **16**, 898−904 (2017).
7. Zhang, X., Ezawa, M. & Zhou. Y. Thermally stable magnetic skyrmions in multilayer synthetic antiferromagnetic racetracks. *Phys. Rev. B* **94**, 064406 (2016).

8. Li, Y., Zhao, C. B., Zhang, W., Hoffmann, A. & Novosad, V. Advances in coherent coupling between magnons and acoustic phonons. *APL Mater*. **9**, 060902 (2021).

9. Dreher, L. et al. Surface acoustic wave driven ferromagnetic resonance in nickel thin films: Theory and experiment. *Phys. Rev. B* **86**, 134415 (2012).
