# Supplementary material for: Ordered creation and motion of skyrmions with surface acoustic wave
Source: Nat Commun. 2023 Jul 22;14:4427. doi: 10.1038/s41467-023-40131-1 (PMC10363109; doi:10.1038/s41467-023-40131-1)
Supplement: Supplementary file 3 — Description of Additional Supplementary Files [file 41467_2023_40131_MOESM3_ESM.pdf]

Description of additional supplementary files

Title: Supplementary Video 1

Description: Simulated evolution of the magnetic domain before and after applying SAWs.

Title: Supplementary Video 2

Description: Simulated current-driven motion of the skyrmions with SAWs at $j = 0.75\times 10^{11}$ A m^{-2}.
